# In-hospital cardiac arrest due to pulmonary embolism – Treatment and outcomes in a Swedish cohort study

**DOI:** 10.1016/j.resplu.2021.100178

**Published:** 2021-11-01

**Authors:** Caspar Epstein Henriksson, Johanna Frithiofsson, Samuel Bruchfeld, Emma Bendz, Maria Bruzelius, Therese Djärv

**Affiliations:** a Department of Medicine Solna, Karolinska Institutet, Stockholm, Sweden; b Emergency Department, Karolinska University Hospital, Stockholm, Sweden; c Department of Hematology, Karolinska University Hospital, Stockholm, Sweden

**Keywords:** IHCA, PE, Thrombolysis

## Abstract

**Objectives:**

Pulmonary embolism (PE) constitutes one of the reversible causes of cardiac arrest. The prognosis for PE-related cardiac arrest is poor. Some previous studies have suggested a higher survival rate in patients with PE-related cardiac arrest who receive thrombolysis. No such study has focused on in-hospital cardiac arrests (IHCA).

**Aim:**

To describe the prevalence of PE-related IHCA and the characteristics of those patients, as well as to describe favourable and adverse outcomes after thrombolysis.

**Material and methods:**

All patients ≥ 18 years who experienced an IHCA at Karolinska University Hospital between 2007 and 2020 with PE as the primary cause of IHCA were included. Patients were identified from the Swedish Registry for Cardiopulmonary Resuscitation (SRCR). Data was collected from the SRCR and medical records. The primary outcome was survival to discharge. Secondary outcomes were alive at the end of CPR, major bleeding, and minor bleeding.

**Results:**

Out of 2,128 IHCA patients, 64 (3%) had a PE-related IHCA of whom 16 (25%) had thrombolysis. A significant association was seen between thrombolysis and survival to discharge (44 % vs 8 %, p-value < 0.01). Major bleeding was not seen in any patient.

**Conclusion:**

Pulmonary embolism is an uncommon cause of IHCA, and thrombolysis is often not administered in such patients. Thrombolysis may increase survival to hospital discharge, and among the selected patients treated with thrombolysis in our study, there was no apparent major bleeding.

## Introduction

Resuscitation of in-hospital cardiac arrests (IHCA) is estimated to occur in 290,000 hospitalised patients annually in the US and about 2,700 IHCAs are reported yearly in Sweden, with an overall 30-day survival of 25%.1., 2.

To identify and treat reversible causes is a critical part of advanced life support, but the certainty of the evidence for recommended interventions in cardiac arrest has been described as generally low.3., 4. One established reversible cause is pulmonary embolism (PE), which is the reported cause of approximately 5% of IHCA cases.5., 6. Few studies have assessed treatment for PE given intra-arrest, but some observational studies on out-of-hospital cardiac arrest (OHCA) and PE have shown no difference7., 8. or increased survival among those receiving thrombolysis.9., 10., 11. No previous studies, however, have examined the association between thrombolysis and outcomes in PE-related IHCA. Thus, the role of thrombolysis in PE-related IHCA remains unclear which is a bit remarkable given that pulmonary embolism is included in the 4H4Ts.

Therefore, our aim was to shed light on the incidence and treatment of PE in IHCA as well as to compare outcomes between patients administered and patients not administered thrombolytic therapy.

## Method

### Study design

This hospital-based retrospective cohort study used the Swedish Registry for Cardio-Pulmonary Resuscitation (SRCR) as the main source to identify all IHCAs at Karolinska University Hospital.

### Settings

Karolinska University hospital is one of five large hospitals in Stockholm (home to approximately 2 million people) and has two equally sized sites, 30 km apart: Solna and Huddinge. The Solna site is a level one trauma unit, has neuro- and thoracic surgery units, and provides angiography for ST-elevation myocardial infarctions at all hours. The Huddinge site includes a geriatric ward and relatively fewer intensive care unit (ICU) beds. Karolinska has about 1,300 beds, 108,000 admissions yearly, and 1.8 million patient visits.

### Ethics

All patients surviving their IHCA were asked six months afterwards for informed consent and agreed to participate in the SRCR and ongoing studies based on it. The Regional Ethical Review Board in Stockholm, Sweden approved the study, ref. 2016/2216–31/2.

### Study population

We defined IHCA according to the SRCR12., 13. as *“a hospitalised patient who is unresponsive with apnoea (or agonal, gasping respiration) where CPR and/or defibrillation have been initiated*.” All adult (18 years) patients who had an IHCA between 1 January 2007 and 31 December 2020 at Karolinska University Hospital were included. No adult patients nor specific locations for the IHCA were excluded. In the case of multiple IHCAs, only the first event per year was included.

### Definitions

#### Pulmonary embolism

Out of the study population above, the following patients were screened for inclusion

a) all cases of cardiac arrest with “PE”, “other respiratory failure” or “other thromboembolism” as the recorded cause of IHCA in SRCR and b) all cases with a missing recorded cause in SRCR. Thereafter we performed a manual review of medical records and included all patients as *confirmed PE* if a PE was found on a computed tomography pulmonary angiogram or autopsy or included as *suspected PE* if PE was stated as the primary cause to the IHCA in medical files. Further information on documented diagnostic tools was also gathered: Echocardiographic signs of PE were defined as those included in the ESC guidelines for the diagnosis of PE (mainly signs of right ventricular dysfunction and right heart mobile thrombus).^14^ Right ventricular dilatation and inferior vena cava distention were not accepted as indicative of PE if only present during CA. ECG findings suggestive of PE were defined as newly emerged right bundle branch block, right axis deviation, SI QIII TIII pattern, or right ventricular strain (T wave inversions in right precordial and/or inferior leads).

Cases, where the primary suspected cause of cardiac arrest was not clearly stated in the medical file, were discussed between three authors (CEH, JF, TD) until consensus and then included or excluded (in all 7 cases, of which 4 were included).

#### Thrombolysis

The only thrombolytic drug available within the hospital for pulmonary embolism was alteplase. We defined a time window of maximum +/-24 hours from the IHCA to administration of thrombolytic therapy.

#### Anticoagulant therapy

Anticoagulant therapy before CA was defined as any dose of heparin, low-molecular-weight heparin (LMWH), warfarin with a PT/INR of >2.0, or direct oral coagulants (DOACs) administered no more than 24 hours before and at least 3 hours before CA.

#### Bleeding

Major bleeding was defined in accordance with the International Society on Thrombosis and Haemostasis criteria for non-surgical patients as death from bleeding, intracranial bleeding, intraspinal bleeding, intraarticular bleeding, pericardial bleeding, retroperitoneal bleeding, intramuscular bleeding with compartment syndrome, intraocular bleeding, bleeding requiring transfusion of two or more units of whole blood or red blood cells, or a haemoglobin drop of at least 20 g/L.^15^ Minor bleeding was defined as any other bleeding noted in medical records.

### Data collection and categorisation

Patients were identified through the hospital’s reported cases to SRCR, where data on the following variables were collected: sex, age (in years), location of IHCA (patient ward, intermediate care unit, intensive care unit (ICU), angiography lab/operating theatre or other areas including emergency department and radiology department), and first documented heart rhythm (shockable, i.e. VT/VF or non-shockable i.e. PEA/asystole). Thereafter by entering the hospital’s electronic patient record (Take Care version 14.2.9) information on comorbidities (ICD-10 codes available at admission to the hospital and assessed according to the Charlson Comorbidity Index,16., 17., 18., 19. PE, thrombolysis, diagnostics, treatments, and outcome was gathered.

#### Outcome

The primary outcome was survival to hospital discharge as recorded in the SRCR. The electronic patient record is linked to the Swedish total population registry and automatically updated within a maximum delay of three days, which enables a national all-encompassing complete follow-up regarding survival.^20^ Secondary outcomes were alive at the end of CPR (yes/no), change in CPC score between admission and discharge, major bleeding within 24 hours from CA, and minor bleeding within 24 hours from CA.

### Statistical analyses

Continuous and discrete variables were reported as medians with interquartile or total range. Categorical variables were expressed as frequencies and percentages. For comparison of categorical variables, Fisher’s exact test was used. For continuous and discrete variables, the Mann-Whitney U-test was used. A p-value of <0.05 was considered significant. Patients with missing data were left out from analyses including the missing variables, and no data was imputed or estimated. All statistical analyses were made using R (version 4.03).

## Results

**Fig. 1:**
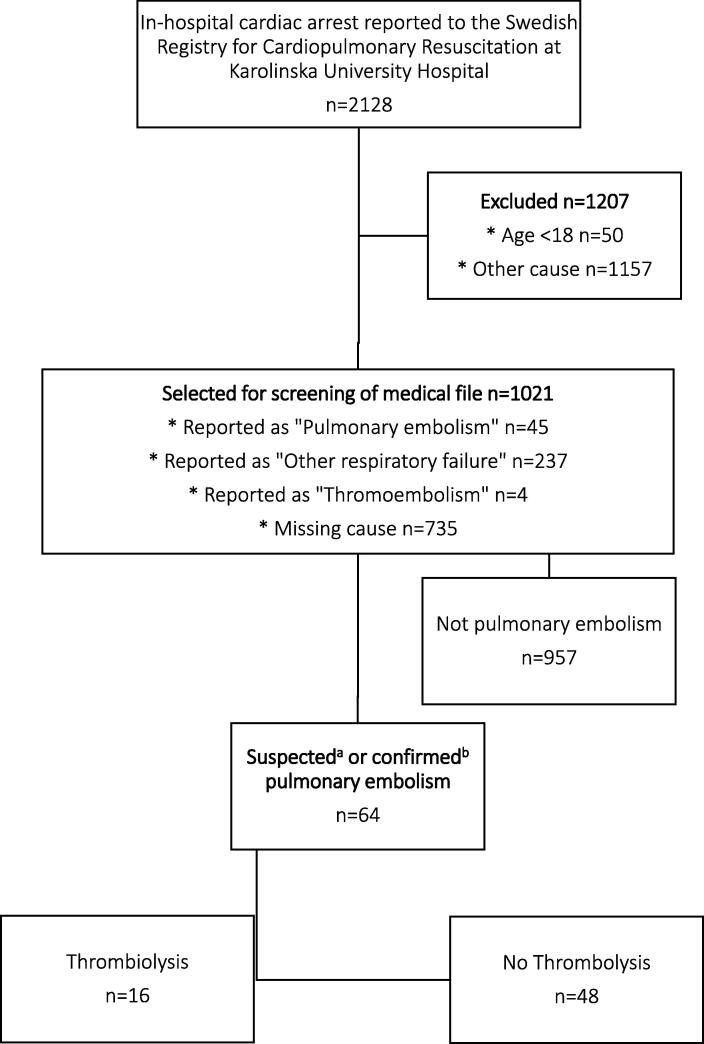
**Flow Chart of Patient Inclusion, In-hospital cardiac arrest at the Karolinska University Hospital 2007**–**2020. ^a^** Stated as the primary suspected cause in medical records or assessed as the most likely primary cause after discussion. **^b^** Confirmed by computed tomography pulmonary angiogram or autopsy.

Out of a total of 2,128 patients who suffered an IHCA at Karolinska during 2007–2020, 1021 (48%) were selected for chart review. After reviewing medical records, 64 (3% out of 2,128) patients with suspected or confirmed PE were included in the final analysis (Fig. 1). Out of the 64, 16 (25%) had thrombolysis.

### Patient characteristics

Of the 64 patients included in the final analysis, 41 (64%) were male and the median age was 71 years. Six (9%) patients had a history of venous thromboembolism, and 13 (20%) had undergone surgery in the 4 weeks leading up to cardiac arrest. Twenty-three patients (36%) had a cancer diagnosis. Twenty (31%) patients had received anticoagulant therapy before CA. The two groups (thrombolysis and no thrombolysis) differed significantly in age, weight, and BMI (Table 1).

**Table 1:** Characteristics and intra-arrest factors in patients having an in-hospital cardiac arrest with suspected pulmonary embolism as the cause at Karolinska University Hospital 2007–2020.

Characteristic	Total *(n = 64)*	Thrombolysis *(n = 16)*	No thrombolysis *(n = 48)*
Age, median (IQR)	71 (59–79)	62 (56–72)	71 (61–83)
Male, no. (%)	41 (64)	9 (56)	32 (67)
BMI, median (IQR)**^a^**	26 (23–31)	31 (29–35)	25 (22–28)
*Medical history*			
Myocardial infarction, no. (%)	9 (14)	2 (13)	7 (15)
Congestive heart failure, no. (%)	9 (14)	2 (13)	7 (15)
Chronic pulmonary disease, no. (%)	12 (19)	2 (13)	10 (21)
Diabetes, no. (%)	16 (25)	5 (31)	11 (23)
Renal disease, no. (%)	7 (11)	0 (0)	7 (15)
Malignancy, no. (%)	23 (36)	4 (25)	19 (40)
Metastatic malignancy, no. (%)	8 (13)	2 (13)	6 (13)
Charlson Comorbidity Index, median (IQR)	2 (0–4)	1.5 (0–4)	2 (0–4)
History of venous thromboembolism, no. (%)	6 (9.4)	1 (6.3)	5 (10)
Surgery (past 4 weeks), no. (%)	13 (20)	2 (13)	11 (23)
Venous thromboembolism the reason for hospital admission, no. (%)	22 (34)	9 (56)	13 (27)
Anticoagulant therapy before cardiac arrest, no. (%)^b^	20 (31)	4 (25)	16 (33)
Days from admission to cardiac arrest, median (IQR)	2 (0.75–7.3)	1.5 (0–4)	2 (0.75–4)
*Circumstances of cardiac arrest*			
*Location*			
ICU/IMCU/CCU, no. (%)	11 (17)	7 (44)	4 (8.3)
Emergency room, no. (%)	7 (11)	3 (19)	4 (8.3)
Ordinary ward, no. (%)	38 (59)	5 (31)	33 (69)
Catheterisation lab/operating room, no. (%)	3 (5.9)	0 (0)	3 (6.3)
Other, no. (%)	5 (7.8)	1 (6.3)	4 (8.3)
ECG monitoring, no. (%)	22 (34)	9 (56)	13 (27)
Witnessed, no. (%)	54 (84)	16 (100)	38 (79)
Arrest during daytime hours (8 AM to 8 PM)	36 (56)	10 (63)	26 (54)
*Initial rhythm***^c^**			
PEA, no. (%)	30 (47)	10 (63)	20 (42)
Asystole, no. (%)	26 (40)	3 (19)	23 (48)
VF/VT, no. (%)	0 (0)	0 (0)	0 (0)
*Treatment of cardiac arrest*			
Epinephrine, no. (%)**^d^**	50 (78)	12 (75)	38 (79)
Antiarrhythmic, no. (%)	8 (13)	3 (18)	5 (10)
Defibrillations, no. (%)	12 (19)	4 (25)	8 (17)
Intubation, no. (%)**^e^**	46 (72)	10 (63)	36 (75)
Time to ROSC, median (IQR)**^f^**	9 (4.5–30)	27 (4.8–45)	6 (4.5–20)

b Any dose of heparin, low-molecular weight heparin (LMWH), warfarin with a PT/INR of > 2.0, or direct oral coagulants (DOACs) administered no more than 24 hours before and at least 3 hours before CA. Data missing for **^a^** 18 patients, **^c^** 8 patients, **^d^** 1 patient, **^e^** 2 patients, **^f^** 3 patients. Abbreviations: CCU – cardiac critical care unit, ICU – intensive care unit, IMCU – intermediate care unit, IQR – interquartile range, PEA – pulseless electrical activity, ROSC – return of spontaneous circulation, VF – ventricular fibrillation, VT – ventricular tachycardia.

### Intra-arrest factors

Most patients suffered their cardiac arrest in wards (n = 38, 59%), a third were ECG monitored and the vast majority were witnessed (Table 1). The initial rhythm was PEA in 30 (47%) patients and asystole in 26 (40%). No patients had an initial shockable rhythm, but 8 (13%) were treated with an antiarrhythmic agent and 12 (19%) were defibrillated. Most patients had epinephrine and were intubated, even if many had short durations of the resuscitation attempt (Table 1). There were significant differences between the two groups (thrombolysis and no thrombolysis) in location and ECG monitoring (Table 1).

### Diagnosis of pulmonary embolism

The diagnosis of PE was confirmed by a computed tomography pulmonary angiogram in 31 (48%) patients (Table 2). Autopsy confirmed the diagnosis in 21 (33%) patients. Two patients had both a computed tomography pulmonary angiogram and an autopsy. In 8 (8%) patients who neither had a computed tomography pulmonary angiogram nor an autopsy, echocardiographic signs of PE were recorded. In 7 (11%) patients, one of whom had thrombolysis, the diagnosis was clinical with no recorded echocardiography, computed tomography pulmonary angiogram, or autopsy. Thirty-eight patients (59%) had a recorded ECG around the time of arrest, of whom 14 (37%) had ECG findings indicative of PE (Table 2).

**Table 2:** Details regarding the diagnosis of pulmonary embolism as the cause for an in-hospital cardiac arrest at Karolinska University Hospital 2007–2020.

Variable	Total (n = 64)	Thrombolysis (n = 16)	No Thrombolysis (n = 48)	P-value
Basis for PE diagnosis				
Clinical only, no. (%)	7 (11)	1 (6.3)	6 (13)	0.32
Echo signs of PEa, but no CTPA or autopsy, no. (%)	8 (12.5)	4 (25)	4 (8.33)	0.01
CTPA, no. (%)	31 (48.4)	9 (56.3)	22 (45.8)	0.57
Autopsy, no. (%)	21 (32.8)	4 (25)	17 (35.4)	0.55
CTPA or autopsy, no. (%)	49 (76.6)	11 (68.8)	38 (79.2)	0.50
ECG recorded, no. (%)	38 (59.4)	14 (87.5)	24 (50)	0.01
ECG findings suggestive of PEb, no. (%)	14 (36.8)	5 (35.7)	9 (37.5)	1

^a^ Right ventricular dilatation in all cases, and right heart mobile thrombus in one case. ^b^ % of patients with recorded ECG. Abbreviations: CTPA – computed tomography pulmonary angiogram, PE – pulmonary embolism.

### Features of thrombolysis

Out of the 16 patients who were administered thrombolysis, 3 (19%) were given the first dose before CA (Table 3). In all, 10 (63%) were given the first dose during CA, and 3 (19%) after ROSC. One patient was administered the first dose of thrombolysis 14 hours after ROSC. All other patients were given the first dose within 2 hours before CA or after ROSC. The given bolus dose was stated in medical records for 11 patients. Of those, 9 patients received 10 mg, one received two 10 mg boluses, and one received 50 mg. Out of the 48 patients who were not administered thrombolysis, PE was suspected by the clinicians according to the medical files from the time of the cardiac arrest for 28 but among these, the reason for not giving thrombolysis was specified only in 6 (21%) patients.

**Table 3:** Features of thrombolysis among patients with pulmonary embolism as the cause for an in-hospital cardiac arrest at Karolinska University Hospital 2007–2020.

Patient	Thrombolysis initiated	Bolus dose	Infusion	Alive at the end of CPR	Survival to discharge	Major bleeding	Minor bleeding	Autopsy
1	After ROSC	10 mg	90 mg, 2 hours	Yes	Yes	No	Yes	–
2	During cardiac arrest	10 mg	No	Yes	Yes	No	Yes	–
3	120 min before cardiac arrest	10 mg	Yes, unknown dose	Yes	Yes	No	No	–
4	140 min before cardiac arrest	10 mg	90 mg, 2 hours	Yes	Yes	No	Yes	–
5	After ROSC	10 mg	90 mg, 2 hours	Yes	Yes	No	No	–
6	During cardiac arrest	10 mg	90 mg, 2 hours	Yes	Yes	No	No	–
7	During cardiac arrest	Missing	Yes, unknown dose	Yes	Yes	No	No	–
8	14 hours after ROSC	Missing	60 mg, 2 hours	Yes	No	No	Yes	No
9	During cardiac arrest	10 mg	15 mg	Yes	No	No	No	Yes
10	During cardiac arrest	20 mg	80 mg	Yes	No	No	No	No
11	During cardiac arrest	Missing	Yes, unknown dose	No	No	No	No	Yes
12	<10 minutes before cardiac arrest	10 mg	90 mg, 2 hours	No	No	No	No	Yes
13	During cardiac arrest	10 mg	No	No	No	No	No	No
14	During cardiac arrest	Missing	Missing	No	No	No	No	Yes
15	During cardiac arrest	50 mg	Yes, unknown dose	No	No	No	Yes	Yes
16	During cardiac arrest	Missing	Missing	No	No	No	No	No

Abbreviations: ROSC – return of spontaneous circulation.

### Survival

Eleven (17 % out of 64) patients survived to hospital discharge. Of those, 7 had been administered thrombolysis, and 4 had not (Table 4). This difference was significant (p-value < 0.01). In all, 22 (34%) patients were alive at the end of CPR. Regarding bleeding, 16 patients were alive to be followed for the entire defined period of 24 hours. Out of the 9 deceased patients, 5 underwent autopsy. Major bleeding was not described in any patient. Minor bleeding was reported in 7 (11%) patients. Of those, 5 had been administered thrombolysis. In the thrombolysis group, minor bleeding presented as haematuria in 2 cases, mucosal bleeding in 2 cases, and spontaneous hematomas on the arms and hands in one case. Among patients who were not administered thrombolysis, minor bleeding presented as one case of rectal bleeding (most likely from haemorrhoid) and one case of intratumoral bleeding (found during autopsy).

**Table 4:** Outcomes among patients with pulmonary embolism as the cause for an in-hospital cardiac arrest at Karolinska University Hospital 2007–2020.

Outcome	Total (n = 64) *No./total no. (%)*	Thrombolysis (n = 16) *No./total no. (%)*	No thrombolysis (n = 48) *No./total no. (%)*	P-value
*Primary outcome*				
Survival to hospital discharge	11/64 (17)	7/16 (44)	4/48 (8.3)	<0.01
*Secondary outcomes*				
Alive at end of CPR	22/64 (34)	7/13 (54)	12/48 (25)	0.05
Major bleeding < 24 hours^a^	0/64 (0)	0/16 (0)	0/48 (0)	–
Minor bleeding < 24 hours^a^	7/64 (11)	5/16 (31)	2/48 (4.2)	<0.01
CPC score, discharge compared to admission**^b^**				
Lower	0/11 (0)	0/7 (0)	0/4 (0)	–
Same	8/11 (73)	6/7 (86)	2/4 (50)	0.49
Higher	3/11 (27)	1/7 (14)	2/4 (50)	–
CPC score at discharge, median (range)	1 (1–3)	1 (1–3)	3 (1–3)	0.11
CPC 1–2	7/11	6/7	1/4	
CPC 3	4/11	1/7	3/4	

a 24 hours from cardiac arrest. **^b^** For patients who survived to discharge. Abbreviations: CPC – Cerebral Performance Category.

Regarding neurological outcome, 8 (73% of survivors) patients had the same CPC score at admission and discharge. In all, 3 (27%) had a higher CPC score at discharge, of those, one had been administered thrombolysis. No patients had a lower CPC score at discharge.

## Discussion

This cohort study is one of few published studies on PE-related cardiac arrests and as far as we know the only comparative one on thrombolysis in an IHCA setting. A suspected or confirmed PE was present in 3% of IHCA patients. A quarter received thrombolysis, which was associated with a higher rate of survival to hospital discharge without major bleeding.

The proportion of IHCA cases caused by PE was slightly less than previous reports of 5%.5., 6. It should be noted, however, that the proportion found in our study could be an underestimate due to a low autopsy rate and diagnostic challenges during cardiac arrest resulting in missed cases of PE.

No patients in our study had an initial shockable rhythm, which is similar to previous studies.5., 21. Despite this, several patients were defibrillated or treated with an antiarrhythmic agent suggesting a change of either rhythm or interpretation of the rhythm during the arrest. Within out study, the locations of the cardiac arrest differed and it is huge difference between a cardiac arrest in the ward and in the icu.

We found survival to hospital discharge of 17%, which is better or in line with previous studies in PE and cardiac arrest,9., 11., 21. and the overall survival for non-shockable IHCA in Sweden.^2^

Previous studies7., 9., 11., 21. have reported a higher frequency of thrombolytic therapy than ours. This might relate to our study including only IHCA patients where more information about the patient such as comorbidity and known contraindications might reduce the tendency to administer thrombolytic therapy. It should be pointed out, however, that the reason for not giving thrombolysis in cases where PE was clinically suspected was stated in a low share of cases. Our finding of higher age and lower BMI among those not treated with thrombolysis might suggest a higher degree of morbidity or frailty not captured in our measure of comorbidities. Furthermore, since thrombolysis was given post-ROSC in three patients, our study might include immortal time bias.

A striking difference between our study and previous ones is that major bleeding was not seen in any patient. Previous studies including only OHCA cases, i.e. Janata et al,^11^ Bougouin et al,^9^ and Javaudin et al,^10^ as well as Yousuf et al^7^ (unspecified location) all report cases of major bleeding, but neither study saw a significant association between thrombolysis and major bleeding. Interestingly, the randomized study on thrombolysis versus placebo in OHCA, the TROICA-trial, found more intracranial bleedings in the thrombolysis group.^22^ The absence of major bleeding in our study might in part be explained by a low autopsy rate. Actually, time to ROSC was longer than usual for IHCA^23^ in those receiving thrombolysis, while the short time to ROSC in those not receiving thrombolysis might partly explain why thrombolysis was not given. Our cohort had similar short times to ROSC It is also possible that non-documented contraindications were present in patients with suspected PE not receiving thrombolysis. Nevertheless, our results are consistent with the findings of Summers et al,^8^ who reported no events of major bleeding in 22 patients with PE-related IHCA who had been administered thrombolysis. It should further be noted that cases of minor bleeding in our study were seemingly unrelated to CPR, and not more common in patients where thrombolysis was initiated during CPR than in those where it was initiated before or after.

Major limitations include the retrospective design and small sample size. The biggest challenge regarding sample size, however, is the low incidence of PE-related IHCA.

Our study design also permits the possibility of survivor bias. Patients who survive CPR are probably more likely to be diagnosed correctly with PE and therefore more likely to be administered thrombolysis. Since it is a retrospective, we are not aware of how much of the available information, for exampled a pathological ECG the clinician in charge used intra-arrest. This is underlined by the fact that PE was never suspected while the patient was alive but later confirmed by autopsy, in a substantial share of patients who did not have thrombolysis.

Supplementary data associated with this article can be found, in the online version, at https://doi.org/10.1016/j.resplu.2021.100178.

As in any cohort study investigating therapeutic interventions, some confounding by indication must be suspected. Though not presented in our results because of the small sample size, a logistic regression model was created where adjusting for age and CCI had the expected effect of decreasing the association between thrombolysis and survival (Supplementary Table 1).

**Supplementary Table 1:** 

For outcomes collected from medical records (i.e., major and minor bleeding), researchers were not blinded to the exposure status (thrombolysis or no thrombolysis) of each patient. This could result in observer bias with a higher frequency of bleeding in the thrombolysis group because of a more meticulous screening of medical records. However, the risk of underestimating the frequency of major bleeding ought to have been eliminated, for the most part, by specifying a clear definition beforehand.

For a higher certainty of evidence for the association between thrombolysis and survival, a larger prospective cohort study would be preferable, or optimally a randomized controlled trial. A randomized controlled trial, however, would probably not be ethically feasible. Further larger studies are also needed to determine the optimal dosage regimen for thrombolysis in PE-related CA and the possible association between thrombolysis and neurological outcome.

In conclusion, our results suggest that PE is an uncommon cause of IHCA, and that thrombolysis is often not administered in such patients. Thrombolysis may increase survival to hospital discharge, and among the selected patients treated with thrombolysis in our study, there was no apparent major bleeding.

## Contributorship

All authors of this manuscript have directly participated in the planning, execution, and analyses of the study. All authors have read and approved the final version of the submitted manuscript. There are no directly related manuscripts or abstracts, published or unpublished, by any of the authors of this paper.

**Table:** 

**Detailed author contribution**	CEH	JF	SB	EB	MB	TD
Study concept and design	X	X	X	X	X	X
Acquisition of data	X	X	X			X
Analysis and interpretation of data	X	X	X	X	X	X
Drafting of the manuscript	X	X				X
Critical revision of the manuscript for important intellectual content	X	X	X	X	X	X
Statistical analysis	X	X				
Obtained funding						X
Administrative, technical, or material support					X	X
Study supervision					X	X

### CRediT authorship contribution statement

**Caspar Epstein Henriksson:** Conceptualization, Methodology, Data curation, Writing – original draft. **Johanna Frithiofsson:** Data curation, Writing – original draft. **Samuel Bruchfeld:** Conceptualization, Visualization, Writing – review & editing. **Emma Bendz:** Conceptualization, Writing – review & editing. **Maria Bruzelius:** Conceptualization, Writing – review & editing, Supervision. **Therese Djärv:** Conceptualization, Writing – review & editing, Supervision.
