# Supplementary material for: In-hospital cardiac arrest due to pulmonary embolism – Treatment and outcomes in a Swedish cohort study
Source: Resusc Plus. 2021 Nov 1;8:100178. doi: 10.1016/j.resplu.2021.100178 (PMC8571515; doi:10.1016/j.resplu.2021.100178)
Supplement: Supplementary file 1 [file mmc1.docx]

**Supplementary table 1. Odds ratios for survival among patients with pulmonary embolism as the cause for an in-hospital cardiac arrest at Karolinska University Hospital 2007-2020.**

| **Outcome** | **Thrombolysis (n=16)**  *No./Total No. (%)* | **No thrombolysis (n=48)**  *No./Total No.  (%)* | **Unadjusted OR**  *(95 % CI)* | **P-value** | **Adjusted OR*^a^***  *(95 % CI)* | **P-value** |
| --- | --- | --- | --- | --- | --- | --- |
| Survival to hospital discharge | 7/16 (44) | 4/48 (8.3) | 8.5 (2.2-39) | <.01 | 7.6 (1.5-42) | .02 |
| Alive at end of CPR | 7/13 (54) | 12/48 (25) | 3.5 (0.98-13) | .05 | 3.0 (0.64-14) | .16 |

Odds ratios calculated using logistic regression. **^a^** Adjusted for age, Charlson Comorbidity Index and location (dichotomized to intensive care unit, cardiac critical care unit or intermediate care unit, or other). Abbreviations: OR – odds ratio.
